# Does “POWS” pack a powerful enough punch to predict pulmonary hemorrhage in preterm infants?

**DOI:** 10.1038/s41390-026-04884-x

**Published:** 2026-03-09

**Authors:** Naveed Hussain, Vineet Bhandari

**Affiliations:** 1 Division of Neonatology, Connecticut Children’s, Hartford, CT USA; 2 University of Connecticut School of Medicine, Farmington, CT USA; 3 Division of Neonatology, The Children’s Regional Hospital at Cooper, Cooper University Hospital, Cooper Medical School of Rowan University, Camden, NJ USA

Pulmonary hemorrhage (PH) in very low birth weight (VLBW) infants remains one of neonatology’s most destabilizing and devastating emergencies that is usually associated with a need for emergency resuscitation in the neonatal intensive care unit (NICU).^1–4^ Yet it remains puzzlingly understudied and therefore, scientifically elusive. The clinical syndrome is recognizable (characterized by an acute respiratory decompensation with bloody secretions in the airway/endotracheal tube accompanied by diffuse radiographic changes), but its incidence, severity spectrum, and mechanistic subtypes are inconsistently defined. Consequently, the evidence for its prevention, early recognition or targeted therapy remains scant.^5–7^ The accompanying paper by Lalos et al.^8^ is therefore timely for its specific analytic findings while also spotlighting the broader vulnerability in neonatology of rare, heterogeneous, poorly phenotyped conditions, which are the hardest to study and yet where we need the most rigorous research.

## Neonatology’s central problem: definition before discovery

A recurring barrier to PH research is that the diagnosis often functions as a final common pathway rather than a single disease. In the current study, PH is pragmatically defined as clinical decompensation plus frank-red or blood-tinged airway secretions, with decompensation including hypoxemia/hypercarbia/shock and/or escalation of respiratory support. This definition aligns with real-world charting, but it also highlights why PH remains under-studied and under-standardized: the same bedside picture may reflect pulmonary capillary “stress failure,” pulmonary edema with hemorrhagic conversion, coagulopathy, infection/inflammation, ventilator-associated injury, transitional hemodynamics, or combinations thereof.^9–14^

When the label is imprecise, traditional science struggles. Mechanistic studies blur distinct pathways into a single endpoint, observational risk-factor studies that generate conflicting signals, and clinical trials are difficult to design because eligibility and endpoints drift across sites. Large databases can enlarge sample sizes, but they also magnify misclassification when diagnostic criteria are subjective or inconsistently coded. The result is a paradox: PH can be terrifyingly memorable in the NICU, yet remain under-characterized in neonatal scientific publications.

## Clinical presentation may be a prelude to a “catastrophe”

One of the important takeaways from this study is that the authors not only investigated “What are the early signs of PH?” but also provide insights into the alterations in vital parameters as an indicator for presumptive changes in infant (patho)physiology *before* PH becomes clinically apparent. Using heart rate (HR) and oxygen saturation (SpO₂) data from two level IV NICUs (from 2016 to 2023), they identified 53 PH diagnoses among 1358 VLBW infants and ultimately analyzed 24 matched PH cases and 24 controls with adequate stored vital signs data.

Their central finding is that pulse oximetry warning score (POWS) rises dynamically beginning ~24 h before the PH diagnosis time (T0), while, at a similar time frame, in weight- and age-matched controls, there was no similar trajectory.

From a physiologic perspective, the finding that SpO₂-derived features outperform HR-only features strengthens the biological plausibility of a subacute prelude to PH, i.e., mean SpO₂ declines and SpO₂ variability rises near T0, and HR–SpO₂ cross-correlation shows improved discrimination as the event approaches.

## Pathophysiologic clues: Not a “bolt-from-the-blue” but evolving cardiopulmonary stress

A predictive signal ~24 h before PH reframes the syndrome. A purely instantaneous event (e.g., sudden bleeding without antecedent physiological strain) is harder to reconcile with a sustained rise in a deterioration risk score. Instead, the data supports an alternative conceptual model, i.e., many PH events may represent the terminal phase of a gradually evolving process with progressive impairment in oxygenation and instability culminating in hemorrhage. The authors themselves note that because SpO₂ metrics performed best, one plausible interpretation is detection of pulmonary capillary congestion prior to hemorrhage with progressive impairment in oxygenation.

That shift matters clinically. It does not prove preventability, but it creates a testable hypothesis: if a subset of infants traverses a measurable “PH-susceptible state,” then earlier identification could support earlier reassessment of ventilation strategy (sustained positive end-expiratory pressure or PEEP), coagulation status, infection and hemodynamic evaluation especially for a significant patent ductus arteriosus (PDA) *before* clinical collapse can occur.

## PDA and PH: A dilemma in the era of expectant management

The manuscript also reopens a perennial question: what is the role of a hemodynamically significant PDA (hsPDA) in PH pathophysiology?^15^ The paper mentions the classic mechanism of ductal shunting and pulmonary over circulation leading to congestion and elevated hydrostatic forces on a fragile pulmonary capillary bed while acknowledging the longstanding debate and mixed evidence.

In this study cohort, PDA was documented more often among cases than controls among those with echocardiography (13/18 vs 9/17), but the authors appropriately avoid causal claims because most echocardiograms occurred after PH and hemodynamic significance was not consistently quantifiable. Nevertheless, it is interesting that the timing of PH occurred at a median postnatal age of 61 h, which is in the window when pulmonary vascular resistance is decreasing, and ductal left-to-right shunt may increase.

A 24-h prelude of POWS risk-warning characterized by worsening oxygenation metrics is physiologically compatible with PDA-induced progressive pulmonary congestion in, at least, a subset of infants. This will need to be reconciled with the contemporary guidance of the 2025 American Academy of Pediatrics report that very early/early routine PDA treatment (<72 h or <7–14 days) is not recommended, and that selective treatment may be considered after 2 weeks for a persistent hsPDA.^16^ The implication is not that PH mandates a return to blanket early closure, but that our field urgently needs precision strategies. How do we identify the infants who are on a pulmonary congestion-to-hemorrhage trajectory from the POWS score and determine whether targeted hemodynamic or respiratory interventions can safely alter course?

## Use of predictive analytics for risk stratification – promises and pitfalls

This paper is also timely in the use of “big-data” in NICU decision-making and the use of physiology-based predictive analytics as a dynamic risk stratification strategy. POWS is computed from continuous bedside monitoring of HR and SpO₂ data and its distributional features and interactions, producing an hourly updated estimate of relative risk of deterioration from baseline. A similar approach using machine learning was used by Aly et al. to study risk factor exposure in 12 h. epochs preceding PH.^17^ The approach has obvious appeal for rare conditions such as PH with subtle antecedents where continuous monitoring can detect patterns clinicians cannot reliably discern in real time, especially in VLBW infants where multiple pathologies share common cardio-pulmonary effects. Others have used different machine learning algorithms to predict PH in preterm infants.^18^ As Lalos et al.^8^ have noted, while CRIB-II scores were similar for cases and controls, the baseline POWS scores were slightly higher in those with PH. Thus, an additional potential benefit is the earlier recognition of evolving instability. Such heightened awareness of a critically ill infant would, in theory, allow for creating windows of opportunity for reassessment and targeted intervention in a particular infant, regardless of the etiology of the instability. The large multicenter randomized trial of heart rate characteristics monitoring (HeRO; MPSC, Charlottesville, VA) associated with reduced mortality in VLBW infants is a good example of how this may be further studied for clinical implementation.^19^

However, we need to exert caution for some significant pitfalls to the approach of physiology-based predictive analytics. First, the potential for a high rate of false positives and the resultant alarm fatigue in a population where instability is common and PH is uncommon. Second, POWS is not risk-specific for PH; in fact, it was developed for prediction of sepsis. Third, the potential for ‘over-intervention’ with more tests, transfusions, antibiotics, ventilator changes, or PDA treatment driven by an elevated score without a validated response pathway. Fourth, there is the problem of generalizability in different NICUs with different devices, signal processing, alarm limits, and documentation. The most useful near-term framing may therefore not be ‘algorithmic diagnosis,’ but ‘analytics-triggered evaluation’ where a significant delta score prompts a structured bedside reassessment and confirmatory testing.

## Priorities for future research

This study prompts readdressing some important issues related to PH. First, the need for consensus phenotyping of the condition with the development of a core definition, severity grading and standardized event timings. Second, the need for studying mechanism-linked cohorts with contemporaneous echocardiographic evaluations (for the diagnosis of hsPDA), ventilator metrics (assessment of lung injury), anemia, coagulation and inflammatory biomarkers (indicators of coagulopathy or sepsis), to distinguish biologically coherent subtypes, if any. Third, multicenter validation linked to interventional trials using standard management algorithms.

## Conclusion

The salient contribution of this work may not be clinical, but conceptual. The study challenges the notion that PH is invariably a sudden, unheralded catastrophe. While possibly lacking specificity for predicting PH, POWS demonstrates the presence of a reproducible physiological prelude and therefore offers both mechanistic clues and a potential path toward prevention.
